# Unbiased serum metabolomic analysis in cats with naturally occurring chronic enteropathies before and after medical intervention

**DOI:** 10.1038/s41598-024-57004-2

**Published:** 2024-03-23

**Authors:** Maria Questa, Bart C. Weimer, Oliver Fiehn, Betty Chow, Steve L. Hill, Mark R. Ackermann, Jonathan A. Lidbury, Joerg M. Steiner, Jan S. Suchodolski, Sina Marsilio

**Affiliations:** 1 Department of Medicine and Epidemiology, School of Veterinary Medicine, University of California, Davis, One Shields Avenue, Davis, CA 95616 USA; 2 Department of Population Health and Reproduction, 100K Pathogen Genome Project, University of California School of Veterinary Medicine, University of California, Davis, Davis, CA USA; 3 Veterinary Specialty Hospital, San Diego, CA USA; 4 West Coast Metabolomics Center, University of California Davis, Davis, CA USA; 5 VCA Animal Specialty & Emergency Center, Los Angeles, CA USA; 6 US Department of Agriculture, National Animal Disease Center, Ames, IA USA; 7 Gastrointestinal Laboratory, Texas A&M University, College Station, TX USA

**Keywords:** Gastrointestinal diseases, Immunological disorders, Metabolic disorders, Cancer models, Gastrointestinal cancer, Cancer, Immunology, Systems biology, Diseases, Gastroenterology, Biomarkers, Diagnostic markers

## Abstract

Chronic enteropathies (CE) are common disorders in cats and the differentiation between the two main underlying diseases, inflammatory bowel disease (IBD) and low-grade intestinal T-cell lymphoma (LGITL), can be challenging. Characterization of the serum metabolome could provide further information on alterations of disease-associated metabolic pathways and may identify diagnostic or therapeutic targets. Unbiased metabolomics analysis of serum from 28 cats with CE (14 cats with IBD, 14 cats with LGITL) and 14 healthy controls identified 1,007 named metabolites, of which 129 were significantly different in cats with CE compared to healthy controls at baseline. Random Forest analysis revealed a predictive accuracy of 90% for differentiating controls from cats with chronic enteropathy. Metabolic pathways found to be significantly altered included phospholipids, amino acids, thiamine, and tryptophan metabolism. Several metabolites were found to be significantly different between cats with IBD versus LGITL, including several sphingolipids, phosphatidylcholine 40:7, uridine, pinitol, 3,4-dihydroxybenzoic acid, and glucuronic acid. However, random forest analysis revealed a poor group predictive accuracy of 60% for the differentiation of IBD from LGITL. Of 129 compounds found to be significantly different between healthy cats and cats with CE at baseline, 58 remained different following treatment.

## Introduction

The metabolome is considered to be a biochemical reflection of the state of an organism’s genome, transcriptome or proteome, representing the current phenotypical status^1^. The detection and characterization of metabolic perturbations has been investigated as a tool for the identification of diagnostic, prognostic, and therapeutic biomarkers in people with inflammatory bowel disease (IBD)^2–5^.

Feline CE (FCE) is a common gastrointestinal disorder in cats, especially in the elderly population^6–8^. A recent study found that up to 32% of all client-owned cats eventually die of gastrointestinal related causes^9^ and the prevalence of CE in cats has dramatically risen over the past decades^10^. Feline CE is defined as the presence of gastrointestinal symptoms (e.g., diarrhea, vomiting and weight loss) for more than 3 weeks in the absence of extra-intestinal or infectious causes^11^. The disorder mainly encompasses inflammatory bowel disease (IBD) and small cell lymphoma (LGITL)^12^, both characterized by similar clinical signs and variable degrees of lymphocytic infiltration of the intestinal mucosa with or without epitheliotropism^13–16^. Despite the high prevalence of FCE, its etiology and pathomechanisms remain largely unknown. However, similar to human IBD, environmental factors, the microbiome and metabolome, and perturbations of the immune system are suspected to play a multifactorial role in the genetically susceptible host^11,17^. Progression of IBD to LGITL has previously been suspected, based on the frequent coexistence of inflammatory and neoplastic lesions in cats with LGITL^18–20^. In addition, chronic inflammation is a well described risk factor for oncogenesis and there are several anecdotal reports of cats with LGITL with a previous history of IBD^18–20^.

A recently revised WHO classification of lymphoid neoplasms in humans includes a new class of indolent gastrointestinal T-cell lymphoma termed gastrointestinal T-cell lymphoproliferative disorder (GI-TLPD)^21,22^. Similar to the disease in cats, GI-TLPD is characterized by small lymphocytes within the mucosa with variable epitheliotropism, and LGITL in cats has recently been suggested as a naturally occurring disease model for GI-TLPD in people^13,23^. Alterations of the fecal microbiome and associated metabolome, that mirror changes characteristic of those in human IBD patients, have also previously been described in cats with CE^24,25^. Given the lack of a distinct mechanistic understanding of feline IBD and its possible progression to LGITL, therapy is largely limited to dietary intervention and systemic immunosuppression by treatment with oral corticosteroids or chlorambucil^6,12,26–28^. The identification of serum biomarkers for FCE could be of great value to: (a) identify non-invasive biomarkers for diagnosis and differentiation of IBD from LGITL, (b) allow for deeper mechanistic insights into the pathology of these disorders, and (c) provide targets for future therapeutic interventions. In addition, serum metabolomic analysis may provide further insights into translational aspects of these disorders under the One Health concept.

We hypothesized that cats with CE have serum metabolomic perturbations and that metabolomic profiling can discriminate these from healthy control cats and differentiate cats with IBD from cats with LGITL. We further postulated that metabolic perturbances found in cats with CE resemble those found in human patients with IBD. Lastly, we hypothesized that metabolic changes return to state closer to that of healthy cats following successful medical intervention as defined by a decreased clinical activity score.

## Results

A total of 42 cats were enrolled, consisting of 14 healthy cats and 28 cats with CE. Of the CE cats, 14 presented with IBD and 14 with LGITL. Sufficient serum samples were available for all 14 healthy cats, and from 13 cats with IBD and 13 cats with LGITL before treatment. Post-treatment serum samples were available for 12 cats with IBD and 14 cats with LGITL (Supplementary Table 5). Patient demographic data are presented in Table 1. Age (*p* = 0.950) and sex (*p* = 0.335) demographics were not different between healthy cats and cats with CE. Cats with CE had a significantly lower body weight (*p* = 0.047) and body condition score^29^ (*p* < 0.001) than healthy control cats. Cats with IBD and cats with LGITL did not differ significantly regarding sex (*p* > 0.999), body weight (*p* = 0.578), or body condition score (BCS)^29^ (*p* = 0.757). Cats with CE had a median feline chronic enteropathy activity index (FCEAI)^12^ of 6 (range 2–11). However, the FCEAI did not differ between cats with IBD (median: 6, range 3–11) and cats with LGITL (median: 5, range 2–10; *p* = 0.408) at baseline nor did the modified clinical activity index (IBD median 3, range 2–5; LGITL median 3, range 1–4, *p* = 0.480) (Tables 1, 2). There was no difference between time to follow-up between cats with IBD (median 44 days, range 20–64) and cats with LGITL (median 35.5 days, range 19–86 days) (*p* = 0.607) (Table 1). The modified clinical activity index improved significantly in both groups between enrollment and recheck examination (Table 2) (IBD median 1, range 0–4, *p* = 0.002; LGITL median 1, range 0–4; *p* = 0.031).

**Table 1:** Demographic data of healthy cats and cats with chronic enteropathy.

	Healthy cats	IBD	LGITL	P-value
Number	14	14	14	
Age in years Median (range)	10.5 (3–14)	8 (2–16)	11.5 (7–16)	0.055
Sex	8 FS, 6 MN	5FS, 9 MN	6 FS, 8 MN	0.510
Breeds	9 DSH, 3 DLH, 1 Persian, 1 Burmese	8 DSH, 2 DMH, 1 DLH, 1 Ragdoll, 1 Siamese, 1 mixed breed	10 DSH, 2 Siamese, 1 DMH, 1 DLH	NA
BCS Median (range)	6 (5–9)	4.5 (3–7)	4 (1–9)	0.001
BW in kg Median (range)	5.1 (4.0- 8.6)	4.6 (2.3 – 5.7)	4.6 (2.9–10.5)	0.065
FCEAI	NA	6 (3–11)	5 (2–10)	0.427
Days between follow up (Median, range)	NA	44 (20–64)	35.5 (19–86)	0.607

BCS, body condition score; 1–4, underweight; 5, ideal; 6–9 overweight; BW, body weight; DLH, domestic longhair; DMH, domestic medium hair; DSH, domestic shorthair; FS, female spayed; MN, male neutered; FCEAI, feline chronic enteropathy activity index^12^.

**Table 2:** Calculation of a modified clinical activity index for cats with inflammatory bowel disease (IBD) or low-grade intestinal T-cell lymphoma (LGITL) to compare data before and after treatment.

	Modified clinical activity index before treatment	Modified clinical activity index after treatment	*p* value
IBD	3 (2–5)	1 (0–3)	0.002
LGITL	3 (1–4)	1 (0–4)	0.031
Combined	3 (1–5)	1 (0–4)	< 0.0001

The index includes being underweight (body condition score ≤ 3), reported weight loss (before endoscopy or after start of therapy, respectively), decreased activity, decreased appetite, diarrhea, and vomiting. A score of 1 was assigned as 1 if signs were present or 0 if signs were absent. Paired data was available for 11 cats with IBD and 8 cats with LGITL.

### Differences between healthy cats and CE cats before treatment

**Figure 1:**
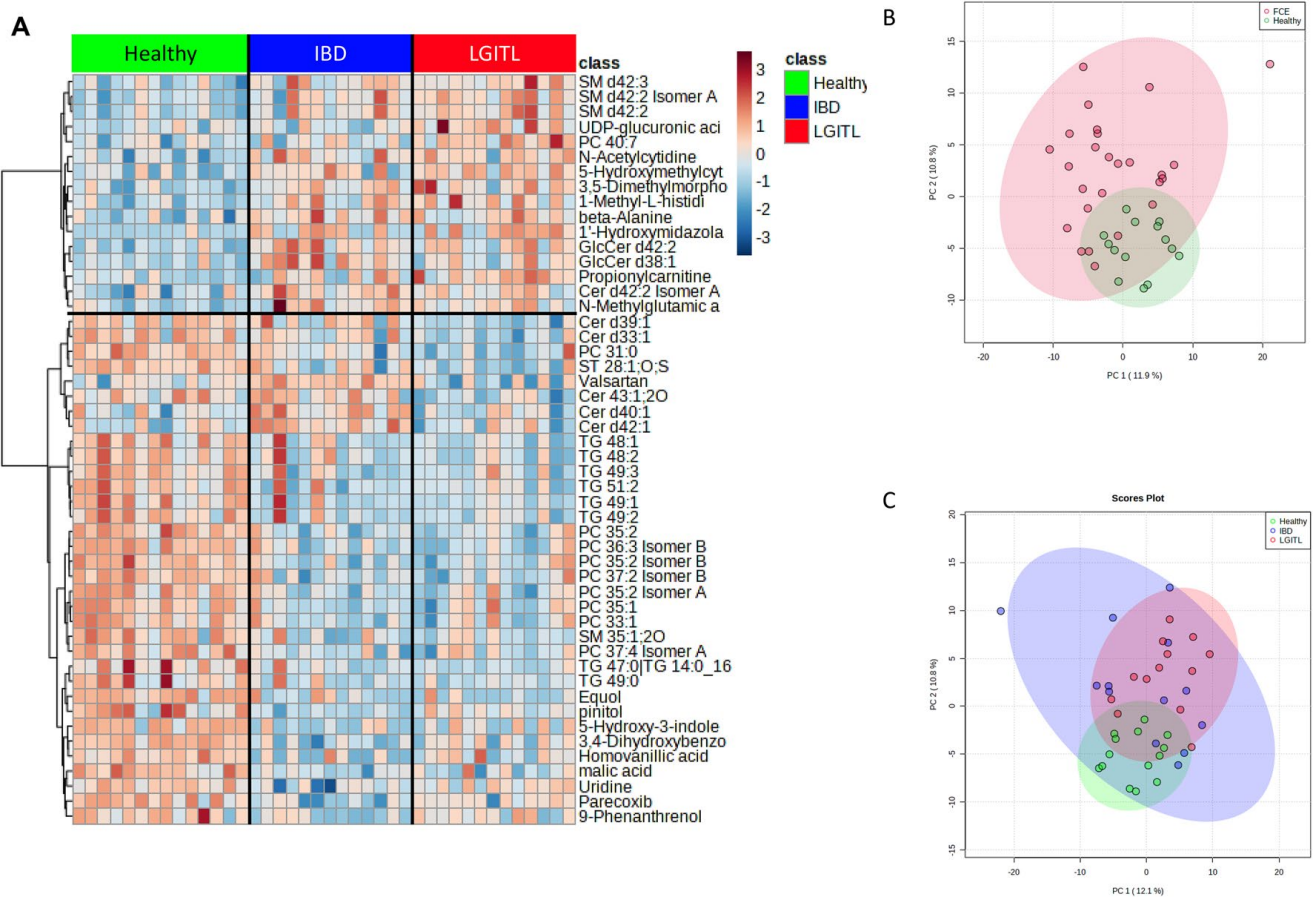
Multivariate analysis of the serum metabolome of healthy cats and cats with chronic enteropathy (CE) at baseline, before treatment. (**A**) Heat map showing metabolites that were significantly different between healthy cats and cats with inflammatory bowel disease (IBD) and low-grade-intestinal T-cell lymphoma (LGITL). Groups are represented by the colored bars at the top of the figure as green (healthy, n = 14), blue (IBD, n = 13), and red (LGITL, n = 13). Clusters can be identified between healthy cats and cats with CE but not between the disease subgroups IBD and LGITL. (**B**) PCA score plots of metabolites in feces from healthy cats (green) and cats with chronic enteropathy (CE, red). (**c**) PCA score plots of metabolites in feces from healthy cats (green), cats with IBD (blue), and cats with LGITL (red). A cluster can be identified for healthy cats but no specific clusters can be seen for the subgroups of IBD and LGITL. Data was normalized to the sample median, log10 scaled, and centered using Pareto scaling.

A total of 1007 named metabolites were detected. Univariate analysis of features selected by Wilcoxon rank-sum test with a threshold of 0.05, and corrected for False Discovery Rate (FDR), found that 129 metabolites differed significantly between control cats and cats with CE (Supplementary Table 1). A volcano plot revealed 41 metabolites to be significantly different with a fold change of 2 or more between control cats and cats with CE (Table 3). Principal Component Analysis (PCA) and a heatmap indicated clustering of the two cohorts (Fig. 1A,B). Random forest analysis revealed class prediction with a 10% out of bag (OOB) error rate, and identified 15 key metabolites by variable importance measure (data not shown), with triglyceride 49:1, lobophysterol C (sterol 28:1;O;S), phosphatidylcholine 35:2, 3,4-dihydroxybenzoic acid and phosphatidylcholine 36:3 being the most important predicted compounds.

**Table 3:** Serum metabolites and their respective pathways significantly altered in cats with chronic enteropathy (CE).

Pathway	Metabolite	FC	log2(FC)	p.adjusted	−LOG10(p)
Organic acids and derivatives					
Carboximidic acids and derivatives	N8-Acetylspermidine	↑2.1296	1.0906	0.018739	1.7273
Organoheterocyclic compounds	Urobilin	↑271.04	8.0823	0.019932	1.7004
Amino acids, peptides and analogues					
Glutamate metabolism	N-Methylglutamic acid	↑2.5481	1.3494	0.0048027	2.3185
Histidine metabolism	1-Methyl-L-histidine	↑2.5045	1.3245	0.0087373	2.0586
	3-Methylhistidine	↑2.1156	1.0811	0.041613	1.3808
Leucine, Lysine, Isoleucine and Valine Metabolism	Val-Lys	↑11.24	3.4906	0.020981	1.6782
	Val-Thr	↓0.3918	− 1.3518	0.021892	1.6597
	N-Methylisoleucine	↑4.8147	2.2674	0.021892	1.6597
	His-Val	↑4.5999	2.2016	0.041613	1.3808
	Lys-Val	↑8.0809	3.0145	0.041613	1.3808
Tryptophan metabolism	5-Hydroxy-3-indoleacetic acid	↓0.1829	− 2.4509	0.0028865	2.5396
Proline metabolism	Proline-hydroxyproline	↑2.8092	1.4902	0.014852	1.8282
Arginine metabolism	Homoarginine	↑2.1825	1.126	0.016864	1.773
Lipids					
Glycerophospholipids	PC 35:2 Isomer A	↓0.45208	− 1.1454	0.0028692	2.5422
Triacylglycerols	TG 48:0	↓0.37119	− 1.4298	0.0058512	2.2328
	TG 48:1	↓0.43156	− 1.2124	0.0031546	2.5011
	TG 49:1	↓0.49948	− 1.0015	0.0014559	2.8369
	TG 50:0	↓0.46563	− 1.1027	0.016183	1.7909
	TG 51:2	↓0.47582	− 1.0715	0.0059618	2.2246
	TG 60:11	↑3.0278	1.5983	0.031851	1.4969
Pyrimidine metabolism, cytidine containing	5-Hydroxymethylcytidine	↑11.261	3.4933	0.0099123	2.0038
	5-Methoxyuridine	↑29.619	4.8885	0.035954	1.4442
Steroids and Steroid Derivatives	Lobophysterol C (Sterol 28:1;O;S)	↓0.47802	− 1.0649	0.013075	1.8836
Hydroxy fatty acids	Mevalonic acid	↑2.771	1.4704	0.035954	1.4442
Drugs and food components					
Drugs	Flunitrazepam	↓0.13768	− 2.8606	0.016864	1.773
	Parecoxib	↓0.15309	− 2.7075	0.0063646	2.1962
	9-Phenanthrenol	↓0.098061	− 3.3502	0.035954	1.4442
	1′-Hydroxymidazolam	↑4.9277	2.3009	0.030435	1.5166
	1′-Hydroxymidazolam beta-D-glucuronide	↑10.664	3.4146	0.020981	1.6782
	Midazolam	↑9.5883	3.2613	0.026251	1.5809
	4-Aminomethylcyclohexanecarboxylic acid	↑14.172	3.825	0.021892	1.6597
Food component/plant	Pinitol	↓0.24563	− 2.0255	0.031851	1.4969
	3,4-Dihydroxybenzoic acid	↓0.12803	− 2.9654	0.00060731	3.2166
	Equol	↓0.30187	− 1.728	0.0046866	2.3291
Pyridines and derivatives					
Pyridines	3-Pyridylacetic acid	↑24.764	4.6301	0.018739	1.7273
Quaternary ammonium salts					
	Carnitine	↑2.5101	1.3278	0.0089358	2.0489
	Propionylcarnitine	↑2.4105	1.2693	0.035954	1.4442
	Isobutyryl-L-carnitine	↑2.517	1.3317	0.013075	1.8836
Vitamins and cofactors					
	Thiamine	↓0.3705	− 1.4324	0.0086415	2.0634
	Tocopherol gamma	↑2.0199	1.0143	0.035954	1.4442
Lactones					
	Glucuronic acid	↑2.1418	1.0988	0.021892	1.6597

↓ indicates downregulation and ↑ indicates upregulation compared with findings in healthy control cats. Fold change was calculated for cats with CE relative to healthy cats.

**Figure 2:**
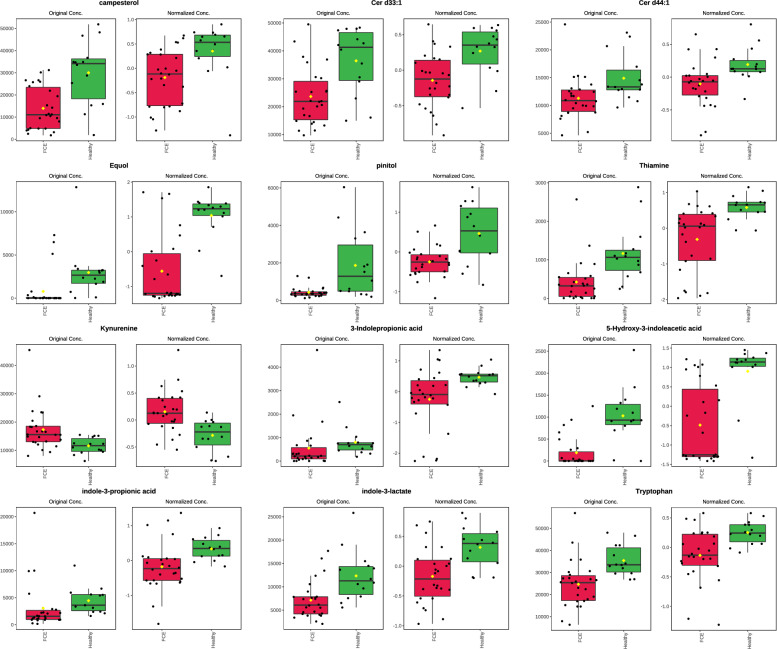
Illustration of results of univariate analysis for select metabolites in healthy cats (Healthy, green box plots) and cats with chronic enteropathy (FCE, red box plots). Compounds including sterols (campesterol) and lipids (ceramides [Cer d33:1, Cer d44:1]), equol, pinitol, and thiamine (Vitamin B1), were decreased in cats with CE compared to healthy cats. Metabolites within the indole/tryptophane/serotonin metabolism showed mixed results. While some compounds such as kynurenine were increased in cats with CE, most indole metabolites such as 3-indolepropionic acid, 5-hydroxy-3-indoleacetic acid, indole-3-propionic acid, and indole-3-lactate, and tryptophane were decreased in cats with CE. Plots on the left reflect comparisons before data normalization, plots on the right reflect comparisons after data normalization. Data were normalized to the sample median, log10 scaled, and centered using Pareto scaling.

Metabolites that were significantly increased in the serum of cats with CE were various amino acids (e.g., alanine, histidine, methionine, lysine, valine), the fatty acids derivative mevalonic acid, and metabolites associated with energy management like carnitine and its derivatives. Compounds that were less abundant in cats with CE compared to healthy cats included vitamins (thiamine [vitamin B1]), fatty acids (triglycerides [TG], ceramides [Cer]), and sterols (campesterol). Compounds within the tryptophan pathway showed mixed results. While some compounds, such as kynurenine, were increased in cats with CE, most indole metabolites such as 5-hydroxy-3-indoleacetic acid (the main metabolite of serotonin), 3-indolepropionic acid, indole-3-propionic acid, and indole-3-lactate, as well as 3-hydroxykynurenine were decreased in cats with CE. However, all compounds within the indole/tryptophan/serotonin pathway had a less than twofold change compared to healthy cats (Fig. 2, Supplementary Table 3).

### Differences between cats with IBD and LGITL

Upon analysis of the CE subgroups IBD and LGITL, one-way ANOVA identified 65 metabolites that were significantly different between all groups (healthy, IBD, LGITL), with 18 compounds significantly different between cats with IBD and cats with LGITL (Table 4).

**Table 4:** Serum metabolites and their respective pathways significantly altered in cats with Inflammatory Bowel Disease (IBD) or Low Grade Intestinal T-Cell Lymphoma (LGITL), compared to healthy control cats, by One-Way ANOVA with Fisher’s LSD posthoc comparisons.

Pathway	Metabolite	f.value	p.value	−LOG10(p)	FDR	Fisher's LSD
Organic acids and derivatives						
Hydroxy acids and derivatives	Malic acid	13.053	5.13E−05	4.2896	0.0069737	Healthy—IBD; Healthy—LGITL
Organic sulfuric acids and derivatives	Homovanillic acid sulfate	7.9129	0.0013775	2.8609	0.028478	Healthy—IBD; Healthy—LGITL
Amino acids, peptides and analogues						
Glutamate metabolism	N-Methylglutamic acid	7.8357	0.0014541	2.8374	0.02881	IBD—Healthy; LGITL—Healthy
Histidine metabolism	1-Methyl-L-histidine	8.231	0.0011038	2.9571	0.026533	IBD—Healthy; LGITL—Healthy
Alanine metabolism	Alanine	7.0474	0.0025514	2.5932	0.04108	IBD—Healthy; LGITL—Healthy
	Beta-Alanine	8.9852	0.00065969	3.1807	0.020912	IBD—Healthy; LGITL—Healthy
Tryptophan metabolism	5-Hydroxy-3-indoleacetic acid	10.891	0.00019092	3.7192	0.013542	Healthy—IBD; Healthy—LGITL
	Kynurenine	7.0351	0.0025741	2.5894	0.04108	IBD—Healthy; LGITL—Healthy
Serine metabolism	Homoserine	7.4135	0.0019608	2.7076	0.035184	IBD—Healthy; LGITL—Healthy
Tyrosine metabolism	Tyrosine	7.0586	0.0025307	2.5968	0.04108	Healthy—LGITL; IBD—LGITL
Lipids						
Sphingolipids	Ceramide d33:1	8.6776	0.00081239	3.0902	0.023401	Healthy—LGITL; IBD—LGITL
	Ceramide d36:1	6.8049	0.0030437	2.5166	0.045701	IBD—Healthy; IBD—LGITL
	Ceramide d39:1	10.036	0.00032948	3.4822	0.015869	Healthy—LGITL; IBD—LGITL
	Ceramide d40:1	8.4296	0.00096253	3.0166	0.024089	IBD—Healthy; IBD—LGITL
	Ceramide d42:1	7.7881	0.0015036	2.8229	0.029183	IBD—Healthy; IBD—LGITL
	Ceramide d42:2 Isomer A	9.2036	0.00056985	3.2442	0.020166	IBD—Healthy; LGITL—Healthy
	Ceramide 43:1;2O	8.2151	0.001116	2.9523	0.026533	Healthy—LGITL; IBD—LGITL
	Ceramide d40:2	6.7906	0.0030755	2.5121	0.045701	Healthy—LGITL; IBD—LGITL
	Glucosylceramide d38:1	8.5611	0.0008796	3.0557	0.0239	IBD—Healthy; IBD—LGITL
	Glucosylceramide d42:2	10.198	0.00029681	3.5275	0.015869	IBD—Healthy; LGITL—Healthy
	Sphingomyelin 35:1;2O	9.3767	0.00050781	3.2943	0.020166	Healthy—IBD; Healthy—LGITL
	Sphingomyelin d42:2	10.553	0.00023637	3.6264	0.014986	IBD—Healthy; LGITL—Healthy
	Sphingomyelin d42:2 Isomer A	11.117	0.00016566	3.7808	0.013542	IBD—Healthy; LGITL—Healthy
	Sphingomyelin d42:3	7.9427	0.001349	2.87	0.028478	IBD—Healthy; LGITL—Healthy
Glycerophospholipids	Phosphatidylcholine 31:0	10.874	0.00019289	3.7147	0.013542	Healthy—IBD; Healthy—LGITL
	Phosphatidylcholine 35:2	15.932	1.02E-05	4.9912	0.004852	Healthy—IBD; Healthy—LGITL
	Phosphatidylcholine 35:2 Isomer A	12.019	9.51E-05	4.0218	0.010555	Healthy—IBD; Healthy—LGITL
	Phosphatidylcholine 35:2 Isomer B	13.7	3.53E-05	4.4526	0.00559	Healthy—IBD; Healthy—LGITL
	Phosphatidylcholine 35:4	7.4241	0.0019461	2.7108	0.035184	Healthy—IBD; Healthy—LGITL
	Phosphatidylcholine 36:3 Isomer B	14.336	2.46E-05	4.6099	0.00559	Healthy—IBD; Healthy—LGITL
	Phosphatidylcholine 37:2 Isomer B	11.938	9.99E-05	4.0005	0.010555	Healthy—IBD; Healthy—LGITL
	Phosphatidylcholine 37:4 Isomer A	9.224	0.00056215	3.2501	0.020166	Healthy—IBD; Healthy—LGITL
	Phosphatidylcholine 40:7	8.0118	0.0012855	2.8909	0.027784	LGITL—Healthy; LGITL—IBD
	Phosphatidylcholine 35:1	9.085	0.00061691	3.2098	0.02023	Healthy—IBD; Healthy—LGITL
	Phosphatidylcholine 33:1	8.1448	0.0011718	2.9311	0.02718	Healthy—IBD; Healthy—LGITL
	Phosphatidylcholine 33:0	7.5296	0.0018053	2.7435	0.033663	Healthy—IBD; Healthy—LGITL
	Phosphatidylcholine 17:0	7.3703	0.0020224	2.6941	0.035616	Healthy—IBD; Healthy—LGITL
	Phosphatidylcholine 31:1	7.0257	0.0025918	2.5864	0.04108	Healthy—IBD; Healthy—LGITL
Triacylglycerols	Triglyceride 48:1	9.7935	0.00038591	3.4135	0.017476	Healthy—IBD; Healthy—LGITL
	Triglyceride 48:2	8.0798	0.001226	2.9115	0.027436	Healthy—IBD; Healthy—LGITL
	Triglyceride 49:1	10.085	0.00031935	3.4957	0.015869	Healthy—IBD; Healthy—LGITL
	Triglyceride 49:2	8.4692	0.00093671	3.0284	0.024076	Healthy—IBD; Healthy—LGITL
	Triglyceride 49:3	8.6395	0.00083375	3.079	0.023401	Healthy—IBD; Healthy—LGITL
	Triglyceride 47:0|Triglyceride 14:0_16:0_17:0	10.065	0.00032338	3.4903	0.015869	Healthy—IBD; Healthy—LGITL
	Triglyceride 49:0	8.4896	0.00092374	3.0344	0.024076	Healthy—IBD; Healthy—LGITL
	Triglyceride 51:2	7.6528	0.0016542	2.7814	0.031464	Healthy—IBD; Healthy—LGITL
	Triglyceride 48:0	7.2865	0.0021474	2.6681	0.03713	Healthy—IBD; Healthy—LGITL
	Triglyceride 47:1|Triglyceride 14:0_15:0_18:1	6.7342	0.0032054	2.4941	0.046897	Healthy—IBD; Healthy—LGITL
Fatty acids and derivatives	(2E)-4-Anilino-4-oxo-2-butenoic acid	6.8684	0.0029058	2.5367	0.044572	IBD—Healthy; LGITL—Healthy
Steroid and Steroid derivatives	ST 28:1;O;S	10.822	0.00019936	3.7004	0.013542	Healthy—IBD; Healthy—LGITL
Nucleotides						
Pyrimidine metabolism, cytidine containing	5-Hydroxymethylcytidine	8.0628	0.0012405	2.9064	0.027436	IBD—Healthy; LGITL—Healthy
	Uridine	9.2852	0.00053966	3.2679	0.020166	Healthy—IBD; LGITL—IBD
	N-Acetylcytidine	8.6344	0.00083664	3.0775	0.023401	IBD—Healthy; LGITL—Healthy
Drugs and food components						
Drugs	3,5-Dimethylmorpholine	9.1034	0.00060936	3.2151	0.02023	IBD—Healthy; LGITL—Healthy
	Parecoxib	13.813	3.31E-05	4.4808	0.00559	Healthy—IBD; Healthy—LGITL; LGITL—IBD
	Midazolam	7.0422	0.002561	2.5916	0.04108	IBD—Healthy; LGITL—Healthy
	1′-Hydroxymidazolam	6.9177	0.0028033	2.5523	0.043704	IBD—Healthy; LGITL—Healthy
	9-Phenanthrenol	7.8773	0.0014123	2.8501	0.028577	Healthy—IBD; LGITL—IBD
	1′-Hydroxymidazolam beta-D-glucuronide	8.8389	0.00072816	3.1378	0.022338	IBD—Healthy; LGITL—Healthy
	Valsartan	10.016	0.00033374	3.4766	0.015869	IBD—Healthy; IBD—LGITL
Food component/plant	Pinitol	11.074	0.00017017	3.7691	0.013542	Healthy—IBD; Healthy—LGITL; LGITL—IBD
	Equol	14.709	1.99E-05	4.7006	0.00559	Healthy—IBD; Healthy—LGITL
	3,4-Dihydroxybenzoic acid	17.268	5.05E-06	5.297	0.0047992	Healthy—IBD; Healthy—LGITL; LGITL—IBD
Organic nitrogen compounds						
Quaternary ammonium salts	Propionylcarnitine	9.1966	0.00057253	3.2422	0.020166	LGITL—Healthy; LGITL—IBD
Lactones						
	Glucuronic acid	9.6605	0.00042104	3.3757	0.0182	LGITL—Healthy; LGITL—IBD

Multivariate analysis revealed distinct clustering of healthy cats, however, cats with IBD and cats with LGITL (Fig. 1C) showed overlapping with no visible clustering. Random forest analysis revealed poor group prediction with an OOB of 40% and class error rates of 69% and 46% for cats with IBD and cats with LGITL, respectively (data not shown).

Amongst compounds significantly different between cats with IBD and LGITL were amino acids (tyrosine), several lipids (ceramides, phosphatidylcholine), DNA metabolites (uridine), and pinitol (Table 4).

### Impact of treatment with corticosteroids and/or chlorambucil on the serum metabolome of cats with CE

**Figure 3:**
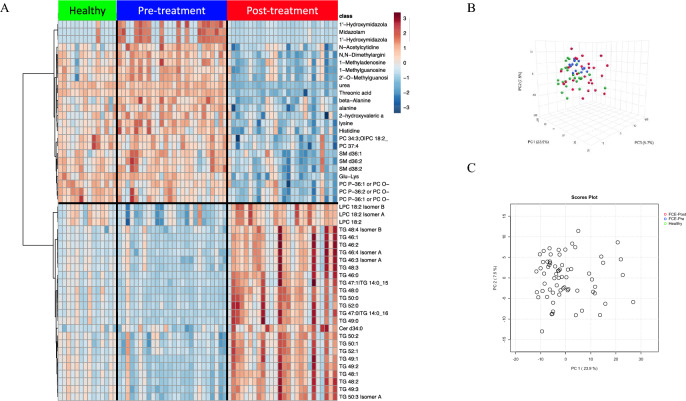
Multivariate analysis of the serum metabolome of cats with chronic enteropathy (CE) before and after 4 weeks of treatment with corticosteroids and/or chlorambucil compared to healthy control cats. (**A**) Heat map showing metabolites that were significantly different between healthy cats and cats CE before (pre-treatment) and after treatment (post-treatment). Groups are represented by the colored bars at the top of the figure as green (healthy, n = 14), blue (pre-treatment n = 26), and red (post-treatment n = 26). While some clustering can be identified, cats with CE do not appear to return to a metabolic profile seen in healthy cats after 4 weeks of treatment. (**B**,**C**) PCA score plots of metabolites in serum from healthy cats (green) and cats with chronic enteropathy before (blue) and after treatment (red). While healthy cats appear to cluster closer together, there is an evident overlap between healthy cats and cats with CE before and after treatment. The data collected after treatment appears to show the most divergence. Data were normalized to the sample median, log10 scaled, and centered using Pareto scaling.

**Figure 4:**
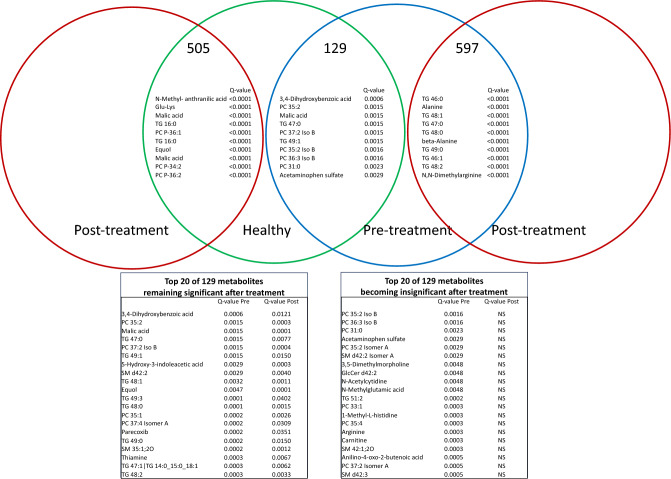
Modified Venn diagram illustrating metabolites found to be significantly different between healthy cats (green circle, Healthy) and cats with chronic enteropathy (CE) before (Pre-treatment) and after medical intervention with corticosteroids and/or chlorambucil (Post-treatment, red circles). The number of metabolites found to be statistically significantly different is indicated on top. The bottom tables show a follow up on the top 20 metabolites that were initially found significantly different between healthy cats and cats with CE. The table on the left indicates the top 20 metabolites that remained statistically significantly different between healthy cats and cats with CE after medical intervention. The table on the right indicates the top 20 metabolites that became non-significant (NS) after medical intervention. Tables indicate the q-values after accounting for the false-discovery rates. Data were normalized to the sample median, log10 scaled, and centered using Pareto scaling.

The serum metabolome of cats with CE before and after medical intervention with corticosteroids and/or chlorambucil differed significantly as seen in the heatmap and PCA plots (Fig. 3). Univariate analysis identified 505 compounds that were significantly different between cats with CE compared to control cats (Fig. 4, Supplementary Table 4) after treatment. A paired analysis within the group of CE cats showed 597 compounds to be significantly different after treatment compared to before treatment (Supplementary Table 6). Of 129 compounds found significantly different between healthy cats and cats with CE before treatment, 58 remained significantly different after treatment (Fig. 4, Supplementary Table 1).

## Discussion

Our study identified significant shifts of the serum metabolome in cats with CE, when compared to healthy control cats before as well as after 4 weeks of treatment with corticosteroids and/or chlorambucil. Multivariate analyses, including hierarchical clustering and PCA, demonstrated clustering of healthy cats and cats with CE. Random forest analysis demonstrated that there is a good group prediction with a low classification error (OOB) of 10%. The results in this study also compared favorably to findings in one of our previous studies investigating fecal metabolomics in a similar cat population^24^. In that study, random forest analysis predicted an OOB of 16.7% for the differentiation of healthy cats from cats with CE based on fecal metabolites^24^.

Studies in human patients have reported similar results in serum or plasma samples with metabolites involving the energy metabolism and amino acids (e.g., lysine and proline) to be altered in patients with IBD compared to healthy controls^30–33^. A previous study also investigated the discriminatory power of serum metabolomics to differentiate various forms of IBD (e.g., Crohn’s disease from ulcerative colitis) in people^34^. The study found metabolites within amino acids (e.g., alanine and citrulline) and lipid metabolism such as phospholipids (phosphatidylcholines) could differentiate Crohn’s disease from ulcerative colitis^35^. Similarly, our study found amino acids (tyrosine) and several lipids (ceramides, phosphatidylcholine) to differentiate IBD from LGITL in cats with CE.

However, while our study identified some metabolites with discriminatory power, multivariate analysis revealed poor group prediction with an OOB of 40% and class error rates of 69% and 46% for the differentiation of IBD from LGITL. This is consistent with results in humans^34,35^ and with previous results from the evaluation of the fecal microbiome and metabolome analysis in cats with IBD and LGITL, which demonstrated significant overlap between groups^24,25^. While disruptions of the mucosal barrier by inflammatory or neoplastic infiltrates are caused by different cellular changes/lesions, barrier disruption by either means could result in similar metabolic consequences and thus the significant overlap between groups.

Following 4 weeks of standard medical treatment with prednisolone or chlorambucil, our study did not indicate a return of the serum metabolome to a healthy state, i.e., the state close to that of healthy control cats. Instead, various metabolic changes between healthy cats and cats with CE following treatment were found, including decreased serum concentrations of amino acids such as proline and tryptophan^34^. This may indicate that metabolic perturbances remain even after successful treatment. Alternatively, it is possible that the treatment itself caused some metabolic derangements or a 4-week follow-up period may have been too short to detect shifts towards a metabolic profile that resembles that of healthy control cats. A previous study investigated changes in the serum metabolome of human patients with IBD following treatment with infliximab (anti-tumor necrosis factor-α monoclonal antibody)^34^. Similar to our results, the serum metabolome did not indicate a return to a healthy state, i.e., the state of healthy control patients. Instead, various metabolic changes between healthy patients and patients with IBD following treatment were described, including decreased serum concentrations of amino acids such as proline and tryptophan^34^.

Based on univariate analysis, we found vitamins (thiamine [vitamin D], tocopherol gamma [vitamin E]), amino acids (tyrosine, derivates of the branched amino acids leucine, isoleucine, and valine) and various lipids including triglycerides, sphingolipids, and phosphatidylcholines, as well as sterols (e.g., campesterol) to be decreased in cats with CE. Chronic gastrointestinal disease and malnutrition can result in various vitamin deficiencies including vitamins in the B, and D family in cats^15,36–39^ and humans^40–43^, and thiamine deficiency has been associated with several gastrointestinal signs, including as nausea, vomiting, and abdominal pain, as well as neurological deficits and fatigue^39,44^. Supplementation with high doses of thiamine have been shown to resolve chronic fatigue in human patients with IBD^42,43^. Lethargy is one of the most common clinical signs in cats with CE^15,45^ and hence it appears possible that lethargy may be partially attributable to a thiamine deficiency. Consistent with our results, previous studies found vitamin E (tocopherol alpha or gamma) to be decreased in serum or plasma samples from humans^46^ and dogs^47,48^ with chronic enteropathies. In addition, a recent study from our group found increased levels of tocopherol and its derivates in feces of a similar cohort of cats with CE^24^. These results may indicate increased intestinal loss and malabsorption of vitamin E in the chronically inflamed gut. Tocopherol alpha and gamma have antioxidant and anti-inflammatory properties and serve as surrogate markers of an organisms’ systemic oxidative status. For instance, studies in dextran sulfate sodium (DSS)-induced colitis in mice have shown tocopherol alpha and gamma to improve intestinal barrier function and induce favorable changes in the gut microbiota^49^. A clinical pilot trial in human patients with ulcerative colitis showed significantly improved clinical activity indices following rectal administration of d-alpha tocopherol^50^. Further studies on the clinical and metabolic effects of vitamin supplementation such as vitamins B, D, and E in cats with CE are needed.

Phosphatidylcholines are a class of phospholipids that are integral part of cell membranes with anti-inflammatory properties^51^. In addition, phosphatidylcholines are part of the intestinal mucous layer and play an important role in the prevention of microbial invasion and translocation^52,53^. Patients with IBD frequently have disruptions of the gut barrier as well as intestinal and systemic inflammation^54^. Phosphatidylcholines have previously been reported to be decreased in the intestinal mucus of patients with ulcerative colitis^55,56^. Both upregulation of phosphatidylcholine hydrolyzing enzyme phospholipase A2 and downregulation of phosphatidylcholine synthase have been documented in human patients and rodent models of IBD and were proposed to be underlying mechanisms of phosphatidylcholine-deficiency^57,58^. Supplementation of phosphatidylcholine in humans has been shown to have anti-inflammatory effects in patients with IBD^51,59^ and improve clinical and endoscopic outcomes, histologic activity, and quality of life in patients with ulcerative colitis^59,60^. Hence, a link has previously been established between phosphatidylcholine deficiency and intestinal inflammation or, possibly, IBD. As previously mentioned, phosphatidylcholines were found to be decreased in cats with CE, compared to healthy controls.

Our data also indicates a decreased abundance of several sphingolipids such as ceramides. This is consistent with results of a previous study of this group documenting increased concentrations of ceramides in fecal samples of cats with CE^24^. Results imply a possible loss of ceramides via intestinal inflammation and a ‘leaky gut’. The role of sphingolipids in IBD is complex and not fully understood. Besides being an integral part of cellular membranes, sphingolipids such as ceramides play a role as cell signaling molecules and modulation of immune responses^61^. Both pro- and anti-inflammatory sphingolipids and ceramides have been described and the conversion of ceramides into different sphingolipids in conjunction with the microenvironment appears to ultimately determine the immune response^61,62^. In addition, the gut microbiota plays a part and some sphingolipids are even be microbiome-derived^63,64^.

Amino acids have previously been investigated as possible diagnostic and therapeutic targets in human patients with IBD. Our study revealed global perturbations within the amino acids metabolism, consistent with previous studies in humans^5^, dogs^65–67^ and cats^24^ with CE.

In accordance with our results, recent studies in dogs with CE found decreased serum or plasma concentrations of several amino acids including tryptophane and tyrosine^65–67^. Decreased concentrations of serum or plasma amino acids may indicate intensified protein catabolism and/or decreased intestinal absorption due to inflammatory changes.

Tryptophan and its related metabolites serotonin, kynurenine, and indole are considered essential for the maintenance of intestinal homeostasis as well as integral signaling molecules of the gut-brain-axis. The amino acid tryptophan is metabolized through 3 different pathways, the indole pathway, the kynurenine pathway, and the serotonin pathway. While primarily known for its role as a neurotransmitter in the brain, 90% of the body’s serotonin production is facilitated by enterochromaffin cells of the mucosal epithelium and mast cells through the conversion of tryptophan via the rate-limiting enzyme tryptophan hydroxylase 1^68^. In addition, several members of the gut microbiome have been documented to produce serotonin^69^. Serotonin regulates intestinal functions including motility, secretion, and absorption through autocrine, paracrine, and endocrine signaling^70–72^. Kynurenine and quinolinic acid are the main products of tryptophan catabolism. Both molecules exert immunomodulatory functions in the gut through aryl hydrocarbon receptors (AhRs) and in the brain through interaction with astrocytes and microglia^72^. The conversion of tryptophan to metabolites within the indole family is solely facilitated by members of the gut microbiota via the expression of tryptophanase (*Clostridium, Burkholderia, Streptomyces, Pseudomonas, Bacillus*^73^) or tryptophan decarboxylases (*Clostridium, Ruminococcus, Blautia, Lactobacillus*^74^)^75,76^.

Microbial-derived tryptophan metabolites, such as indole and some of its downstream metabolites indole-3-acetate (IAA) and tryptamine, are signaling molecules for AHRs and peroxisome proliferator-activated receptor (PPAR)^77^ and have been shown to attenuate intestinal inflammation^78^. AhR signaling regulates intestinal mucosal homeostasis by acting on innate and adaptive immune cells as well as on epithelial renewal and mucosal barrier function^79^. Alterations of the tryptophan pathways and tryptophan deficiency have long been associated with the devolvement or exacerbation of intestinal inflammation in human patients with IBD^80^ or those with irritable bowel syndrome (IBS)^69,70^ as well as in dogs^67,81,82^ and cats^24,83^ with CE. Similarly, our study identified several metabolites within the tryptophan pathway to be altered in serum samples from cats with CE including tryptophan, 3-indolproprionic acid, indole-3-proprionic acid, indole-3-lactate, 5-hydroxy-3-indolacetic acid (all decreased), and kynurenine (increased), albeit not all compounds met the threshold of a twofold change in abundance. Interestingly, we previously reported the opposite for findings in the fecal metabolome where the abundance of metabolites within the tryptophan family increased, hinting to a possible loss of tryptophan and its metabolites through damaged mucosal barrier^24^. These findings may imply that tryptophan and its related metabolites might be valuable targets for the development of diagnostic tests or therapeutic interventions.

Similar to a previous study in dogs^66^, we found derivates of branched amino acids (BAAs) including leucine, isoleucine, and valine to be increased in serum samples of cats with CE. BAAs have been shown to play a pivotal role in chronic inflammatory conditions in humans including metabolic syndrome, obesity, cardiovascular disease, and diabetes mellitus and have been investigated as biomarkers of disease and risk prediction^84^. However, other studies in dogs^65^ and humans^85^ did find either no differences or even decreased serum or plasma BBA concentrations in patients with CE compared to healthy controls. It is unclear whether these discrepancies may reflect differences related to species, study populations or diagnostic assays. Targeted studies generating quantitative data are required to verify findings.

Amongst the metabolites with post-hoc analysis significant for all comparisons (Healthy-IBD, Healthy-LGITL, IBD-LGITL) were phosphatidylcholine 40:7, pinitol, and 3,4-dihydroxybenzoic acid (Table 4), all compounds with a described protective effect in intestinal barrier disorders^86–88^. Pinitol is a phytochemical with antioxidant, anti-diabetic, anti-inflammatory and anticancer properties^86,89^. 3,4-dihydroxybenzoic acid (protocatechuic acid) is another plant-derived xenobiotic with antioxidant and anti-inflammatory properties. Both supplementation with pinitol and protocatechuic acid have been shown to alleviate inflammation and clinical signs in rodent models of ulcerative colitis^86,90^. Results are consistent with previous studies, which have found several phytosterols, including campesterol, fucosterol, and stigmasterol to be altered in fecal samples of dogs and cats with CE^24,82,91^.

Results of our study imply that metabolic derangements do not appear to resolve or return to a state closer to the metabolic state of healthy cats (Figs. 3, 4) despite a significant clinical improvement of most cats. While the metabolic profile of healthy cats clusters close together, metabolic profiles of cats with CE before and after treatment with corticosteroids or chlorambucil appear diverse and post-treatment samples do not show a clear cluster. Metabolites and pathways that remained altered even after clinical improvement include phosphatidylcholines, sphingolipids and thiamine. Metabolites showing transition to a non-significant difference include amino acids and their metabolites (arginine, methyl-L-histidine), ceramides (Fig. 4).

This study has several limitations. According to current guidelines, a diagnosis of IBD was made based on chronic signs intestinal disease of at least 3 weeks duration, the absence of known enteropathogens or other causes of signs of gastrointestinal disease, and the histopathologic confirmation of intestinal inflammation^11^. While some cats were on a hypoallergenic diet, we did not exclude dietary-responsive enteropathy in all cats. However, the term dietary-responsive enteropathy is currently ill-defined and no guideline exist in veterinary literature regarding the number, type, or duration of dietary trials that cats should undergo to confirm or reject a diagnosis of dietary-responsive enteropathy. Owner compliance is frequently difficult to ascertain and anecdotally, cats and dogs can fail to respond to one diet but may respond favorably to another. In addition, it is recognized in human medicine, that IBD is an umbrella term for a number of disorders of different etiopathogenesis and many human patients with IBD show complete or partial resolution of clinical signs in response to dietary interventions but are not reclassified as dietary-responsive enteropathy^92–95^.

In addition, we did not perform fecal cultures for known fecal pathogens. However, obligate pathogens usually cause acute but self-limiting diarrhea in cats and histopathological signs of neutrophilic enteritis. Our study only included cats with chronic signs of intestinal disease, diarrhea was rather rare amongst our population, and all cats had either lymphoplasmacytic enteritis or LGITL. Inflammatory lesions can be difficult to distinguish from LGITL in cats with CE and despite additional diagnostic testing including Immunohistochemistry and clonality testing, ambiguous cases may remain^11,15,16,96^. However, as previously described, all available clinical, laboratory, and pathological data were integrated to arrive at a final diagnosis and cases that remained ambiguous were excluded from this study^11,97–100^. Nevertheless, given the lack of a clear gold standard in the differentiation of IBD from LGITL it is possible that some cases may have been misclassified. In addition, this study did not use immunohistochemistry stains such as pSTAT3, pSTAT 5, or Ki-67. A recent study suggested these markers to potentially assist in the differentiation of inflammatory from neoplastic lesions in the intestinal tract of cats with CE. However, the markers have not yet been validated and tested in routine diagnostic settings, nor been endorsed as standard markers by the recent consensus statement on the diagnosis and differentiation of low-grade neoplastic from inflammatory lymphocytic chronic enteropathies in cats^11^.

This study aimed to identify clinically relevant alterations of the serum metabolome and while demographic characteristics of cats were comparable (Table 1) this study did not control for environmental and dietary components. While changes of the metabolome have been described due to exogenous and endogenous factors including genome, diet, and environment, controlling for those factors would likely result in a negative impact on clinical applicability. Recent studies do imply that over-standardization is a main reason for poor reproducibility of pre-clinical trials^101,102^. Although, we cannot rule out that those factors confounded our results, most are in line with findings across different species with spontaneous or induced IBD and thus likely reflect true changes of the fecal metabolome.

Our study did not control for housing, diets, or treatments, and treatments were assigned upon the attending veterinarian’s discretion based on the patients’ clinical requirements. Most cats were housed indoors and fed a variety of different but commercial balanced diets. All cats received corticosteroids and/or chlorambucil in addition to a variety of other treatments. In addition, time to follow-up examination differed between cats. Variabilities in these factors, especially different treatment regimens, could explain the ongoing metabolic alterations. To the author’s knowledge there is no data on the impact of corticosteroids, chlorambucil, and other drugs commonly used for the management of IBD and LGITL in cats available today which would be the prerequisite to understand the sole impact of such treatment on the serum metabolome. However, the scope of this study was to assess whether improved clinical signs correlate with a shift of the serum metabolome to a healthy state. Both, cats with IBD and LGITL improved their clinical activity indices after start of treatment (Table 2). However, metabolic alterations remained. The clinical activity index used in this study has not previously been validated. However, the only previously published clinical index, the FCEAI^12^, has challenges with sufficient comparison of longitudinal data as it includes parameters such as the presence of endoscopic lesions which are usually not available for follow up. In addition, the scoring of 0 to 3 is subjective and varies between clients. Therefore, a modified binary (absent or present) index^15^ based on available data suitable for follow up was used for this study.

Lastly, this study included a limited number of cats which may have introduced errors, specifically type II statistical errors (false negatives). A larger samples size may have allowed to detect more metabolites of significantly different abundance between groups. However, statistical significance does not equal clinical relevance. Unbiased metabolomics studies are typically performed on a limited number of subjects and aim to provide quantitative data (relative changes) of possible biomarker candidates which subsequently need to be verified by targeted assays, ideally in a separate population of cats. While it is likely that a larger sample cohort would have resulted in more significant findings, the scope of this study was exploratory and to establish a direction to further build the basis for future targeted and quantitative studies on clinically relevant biomarkers.

In summary, our study revealed changes of the serum metabolome in cats with CE with involving lipids, amino acids, vitamins, and metabolites within the tryptophane family. Changes were consistent with those of previous studies in other species. Besides diagnostic targets, we have identified several potential therapeutic targets including thiamine, phosphatidylcholine, and phytosterols. Global metabolic perturbances did not resolve after 4 weeks of treatment with corticosteroids and/or chlorambucil, however, the impact of such treatment alone on the serum metabolome remains unclear. Future targeted studies should investigate whether a biomarker or a combination of biomarkers will allow for improved minimal-invasive diagnostic testing or serve as therapeutic targets and markers to determine treatment success.

## Methods

### Study approval and enrollment

Patient examination and sample collection for this prospective study was conducted at the Veterinary Medical Teaching Hospital at Texas A&M University between May 2015 and September 2017. The study protocol was approved by the Texas A&M University Institutional Animal Care and Use Committee (IACUC 2015–0276 CA and IACUC 2014–0369 CA). All experiments were performed in accordance with relevant guidelines and regulation and we confirm that the authors complied with the Animal Research: Reporting of In Vivo Experiments (ARRIVE) guidelines^103^. Cat owners provided written informed consent prior to study enrollment. Cats with clinical signs of chronic enteropathy (n = 28) and control cats (n = 14) were recruited from the hospital population at the Small Animal Hospital of Texas A&M University in College Station, Texas or the Veterinary Specialty Hospital in San Diego, California^24,25^.

The health status of cats in the group considered healthy was verified by an owner questionnaire on general and gastrointestinal health^24,25^. The questionnaire covered the following areas: attitude/activity, appetite, drinking, urination, chronic illnesses, weight loss, vomiting, diarrhea, and treatment with antibiotics, antacids, anti-inflammatory drugs, or steroids^24,25^. In 11 of 14 cats, physical examination and blood testing was available and performed by a single board-certified internist (SM). The body condition score was assessed using a previously established nine-point condition scoring system^29^.

Blood was collected from a peripheral vein or the jugular vein and the following tests were performed: complete blood count (Advia 120, Siemens Medical Solutions USA, Inc., Malvern, PA, USA), serum chemistry profile (Beckmann AU480, Beckman Coulter, Inc., Brea, CA, USA and Vitros 4600, Ortho Clinical Diagnostics, Raritan, NJ, USA), total T4, cobalamin, folate (all measured on Siemens Immulite 2000 xPi, Siemens Medical Solutions USA, Inc., Malvern, PA, USA), feline pancreatic lipase immunoreactivity (fPLI) (Spec fPL™, IDEXX Laboratories Inc., Westbrook, ME, USA), and feline trypsin-like immunoreactivity (fTLI) (Radioimmunoassay, Gastrointestinal Laboratory, Texas A&M University, USA). Cats with gastrointestinal signs (i.e., weight loss, hyporexia, vomiting > 2x/ month, diarrhea) within 6 months prior to enrollment were excluded. In addition, cats with systemic diseases, chronic illnesses or clinically significant laboratory abnormalities were excluded from the study^24,25^. Finally, cats that had received any antibiotics, antacids, anti-inflammatory drugs, or corticosteroids within the past 6 months were excluded^24,25^.

Cats with clinical signs of chronic enteropathy (weight loss, hyporexia, vomiting, diarrhea) of at least 3 weeks duration were eligible for enrollment into the group of cats with chronic enteropathy^11^. Extra-gastrointestinal disease as well as, where indicated, infectious intestinal diseases were excluded based on a complete blood count, serum chemistry profile, total T4 and fecal examination^24,25^. All cats in this group underwent gastro-duodenoscopy and ileo-colonoscopy for diagnostic purposes. Histopathologic examination of hematoxylin and eosin-stained endoscopic formalin-fixed, paraffin-embedded (FFPE) tissue sections was performed by a single board-certified pathologist (MA) specialized in the assessment of small animal gastrointestinal biopsy specimens and blinded to the clinical status of the cats. All samples were assessed according to histopathological standards for the evaluation of intestinal biopsy specimens as previously described by the World Small Animal Veterinary Association Gastrointestinal Standardization Group^24,25^. Cases with a histopathological diagnosis of LGITL or cases where the pathologist was suspicious of an underlying LGITL underwent additional diagnostic testing with immunohistochemistry including CD3 or CD79a staining and PCR for antigen receptor rearrangement testing for diagnostic confirmation^11,97,98^. A final diagnosis of IBD or LGITL was reached upon integration of results from histopathology, immunohistochemistry, and PCR for antigen receptor rearrangement (PARR) based on the current EuroClonality/BIOMED-2 guidelines for interpretation and reporting of Ig/TCR clonality testing in suspected lymphoproliferations^11,97–100^. Cats that had received antibiotics within 4 weeks or corticosteroids within the past 2 weeks prior to sampling were excluded from the study^24,25^.

### Treatment and sample collection

14 healthy cats and 28 cats with CE (14 cats with inflammatory bowel disease (IBD), 14 cats with Low Grade Intestinal T-Cell Lymphoma (LGITL), were enrolled into the study. Clients were asked to return approximately one month after enrollment. However, scheduling of the recheck examination was performed at the attending veterinarian’s discretion, based on patient’s needs, and the client’s availability. All cats received treatment with corticosteroids and/or chlorambucil at various doses. In addition, cats received a variety of treatments at the attending veterinarian’s discretion including antimicrobials, proton-pump inhibitors, probiotics, and cobalamin.

### Metabolite analysis

Metabolites were extracted from serum samples and analyses were performed by the West Coast Metabolomics Center, University of California Davis (Davis, CA, USA), as previously described^104–106^. We then acquired data to report a total of 1,037 metabolites with known chemical structures using four separate assays at the UC Davis West Coast Metabolomics Center. For all assays, NIST SRM1950 standardized plasma, pooled quality controls and blank quality controls were used to validate the precision of compound quantifications. (1) *Primary metabolism* data were acquired by gas chromatography/time-of-flight mass spectrometry (GC-TOF MS). Data validation protocols and quality controls have been extensively described previously^107,108^. Using data processing and mass spectral deconvolution in ChromaTOF 4.0 (Leco, St. Joseph/MI), we detected 170 unique identified chemicals and 286 unknowns with BinBase DB identifiers^109^. Primary metabolism data was normalized to the sum of all annotated metabolites for metabolite i of sample j:

- metabolite_ij,normalized_ = metabolite _ij,raw_/mTIC_j_*mTIC_average_ as given in^107,108,110^.

Data were constrained at s/n > 10 using method blank samples, and checking consistency using pooled quality control samples that were interspersed after each set of 10 cat serum samples.

(2) For *ionic metabolites and biogenic amines*, we have used hydrophilic interaction liquid chromatography (HILIC) on a 6600 Sciex quadrupole-time of flight mass spectrometer (TTOF) in both negative and positive electrospray mode. Thirty internal standards including betaine, choline, taurine, amino acids, acylcarnitines, peptides and methylnicotinamide were used for data normalization and quality control^111–113^. Chromatography was performed on a Waters Acquity UPLC BEH Amide Column (1.7 µm, 2.1 mm × 150 mm) with an acetonitrile/water gradient (10 mM ammonium formiate + 0.125% formic acid buffer) scanning m/z 60–1200 Da and 45 V collision-induced fragmentation energy at 4 spectra per second. Raw data was processed using MS-DIAL v. 4.70 software^114^ that led to annotation of 370 unique metabolites using public mass spectra collected at the MassBank of North America plus NIST20. Annotations were accompanied by MSI level confidence codes^115^. Data quality control was performed by normalizing data to the sum of internal standards, plus data curation using pooled quality control and method blank samples as above. (3) For *lipidomics analyses*, we have performed extractions on 50 ul serum samples using 1 ml of an aqueous methanol/MTBE mixture at -20°C, dried down the lipophilic phase and re-suspended it in methanol/toluene (9:1, v/v) prior to LC–MS/MS analysis^116^. Lipids were separated on a Waters CSH C18 UPLC column (100 × 2.1 mm; 1.7 µm) at 65 °C at a flow-rate of 0.6 mL/min with a gradient from 60:40 (v/v) acetonitrile:water to 90:10 (v/v) isopropanol:acetonitrile^117^. We used both positive and negative electrospray ionization mass spectrometry on an Agilent 6546 mass spectrometer, acquiring MS/MS data at 4 Hz from m/z 50–1700 at 15,000 resolving power (FWHM), using capillary voltage + /− 3 kV and nitrogen gas temperature 325 ºC at 8 L/min. Data was processed in MS-DIAL v. 4.70 software^118^. Recursive data analysis was used to search and replace missing values from raw data files. LipidBlast as part of MassBank of North America was used to identify lipids by accurate mass, MS/MS and retention time matching, using MSI confidence levels as above to annotate 474 unique lipids. Data normalization was performed on 65 internal standards (Splash One kit, Avanti Polar Lipids, Birmingham/AL) in addition to the used of pooled quality control and method blank samples as above. (4) *Targeted analyses of bile acids* was performed on 50 ul serum samples using 150 ul cold acetonitrile/methanol extractions with 12 internal standards for quality control and determination of micromolar concentrations. Data for 23 bile acids were reported using C18 reversed phase liquid chromatography and mass spectrometry on a Sciex 6500 + quadrupole-ion trap (QTRAP) instrument with individually optimized declustering potentials, entrance potentials, collision energies, MS/MS transitions and collision exit potentials for every analyte. Sciex Analyst software was used for peak integration and quantification to selected internal standards.

### Statistical analyses

Patient demographics were compared by the Mann–Whitney or Fisher’s exact test as appropriate using Prism 10.0 (Graph Pad Software, La Jolla, CA). Univariate and multivariate analyses were performed using MetaboAnalyst^119^. Data were median centered, log transformed, and Pareto scaled to achieve normalization. Statistical significance was set at *p* < 0.05, and minimal Fold Change was set at twofold (> 2 or < 0.5). In all cases, data were adjusted using False Discovery Rate and expressed as q-values. Differences between healthy and CE cats were evaluated using paired t-tests, adjusted using False Discovery. Differences between healthy, IBD and LGITL cats were evaluated using the Kruskal-Walis Test, the One-Way ANOVA non-parametric version, and post-hoc Fisher’s LSD Analysis. Multivariate analysis included Principal Component Analysis (PCA), hierarchical clustering with Euclidean distances using the Ward method, and heatmap generation as a visual aid for the dendrogram. Random forest regression analysis was used to evaluate the classification performance of metabolomics.

### Ethical approval

This article does not contain any studies with human participants performed by any of the authors.

### Research involving animal rights

All applicable international, national, and/or institutional guidelines for the care and use of animals were followed.

### Supplementary Information

Supplementary Table 1.

Supplementary Table 2.

Supplementary Table 3.

Supplementary Table 4.

Supplementary Table 5.

Supplementary Table 6.

## Data Availability

The datasets generated and/or analyzed during the current study are available from the corresponding author on reasonable request.
